# On Cold Affusion in Gout

**Published:** 1804-06-01

**Authors:** 


### *To the Editors of the Medical and Physical Journal.*

GENTLEMEN,

ANY thing like vindictive controversy or replications of illiberality or malevolence, ought to be universally deprecated and scouted; but in what manner such have (as Medicus has asserted in your last Journal) tended more to embarrass than to elucidate the science of medicine, demands a more satisfactory proof than mere suggestion.

What has excited controversy of such obnoxious contexture, seems to be the fair relation and detail of certain experiments, or the result of individual practice, upon the salutary operation of cold affusion in arthritic paroxysms. From the recent introduction of this mode of treatment of gout, has arisen a conflict which, although it has little or no claim to candour, nor is it indicative of benevolent interest for public welfare, is certainly incapable of degrading your Journal, embarrassing the science, or sinking the exalted establishment of medical enquiry into contumelious puerility.

I must conclude with Medicus, that theories are very often intended more to convey plausible and ingenious opinions of subtle speculatists, than to lead to a prompt and successful practice; yet I cannot but observe, that whatever may be our deficiences in the knowledge of the operations of the nervous system, there may be good room for cautious experiments and warrantable deviations from general practice, if they are derived from strict analogy, and supported by a successful issue.

It much surprises me, that Medicus, who starts as a reformer of medical discussion, and the strong advocate for the plain and simple detail of all means from the weakest to the most potent, conducive to the cure of disease, should so far forget himself as not only to fall into the defects of which he so much complains in others; but also, that he should depreciate the merits and the good effects derived from from the practice of cold ablution in gouty affection. According to him, the *practice is contrary to all sound experience*; not *one instance,* however, does he record where he observed its pernicious effects. Instead of facts he contents himself with assertions.

If gout be a general disease, (as Medicus believes it to be) why should not local means contribute to the cure, or remove that local affection which is the principal part of the disease; the removal of which, as in many cases of symptomatic affections, might accelerate the progress of a general, or at least temporary cure of the whole? But we know as little concerning the causes of disease in general, especially of gout, or how they induce diseases, as we do of nervous operations; and in how many instances do we endeavour to cure general morbid action by destroying the principal leading symptoms? But the grand question seems now to be, whether the gout be a local or general affection? or whether, after the frequent repetitions of its severity, it may not become catenated with, or form general morbid sympathies? Now, should the gout be originally a general disease, there can be no pathological reason why the practice of local means, which vanquishes the most harrassing symptom, should not induce a salutary change in the diseased movement of the animal machine, and by that process, produce a restoration of its healthy faculties. By the same rule, those diseased sympathies arising from local arthritic affections, should gout be primarily unconnected with the system, may be interrupted, and more salutary, o ]the accustomed healthy action redintegrated by the mere influence of local remedies.

Let the opinions of medical men rest upon either side of the question, no ground can be tenable for uncandid and inefficient declamation; and a stubborn inflexibility in opposing practical and laudible innovation is more injurious to science than the greatest absurdity of the most flimsy hypothesis.

I could adduce as many instances in support of the efficacy of the reduction of temperature in gouty paroxysms, by cold water, as Medicus has supposed instances contraindicative of its use or inadmissibility; but I shall content myself in the relation of a single instance, where the application of cold water was attended with the happiest result. In this case it was not directed by practical experience or legitimate speculation, but by the mere instinctive impulse of the suffering victim.

A super-

A superintendant of a fishery in Newfoundland, labouring under a gouty paroxysm, and, as is usual, strongly advised to remain confined to his room, suffered severely from the pain and great inflammation in one of his feet; but wishing to discharge his official duties, he became determined, against all persuasion, to go to sea in one of the fishing vessels. He silenced all opposition to his resolve, by crying out that his pain and the heat of the affected part were so great, he was confident nothing could abate them but cold; and if that did not relieve him he would as lief die at sea as suffer so much torment in bed. The consequence was, that the poor sufferer was carried to the vessel, in which he put to sea, and was obliged, as is usual, to remain for several hours nearly up to his knees in salt water. In this situation, to the great astonishment of every one present, the pain and inflammation abated, and the man ceased to complain; on his return home he insisted on his wet clothes remaining upon him, and in case of the symptoms returning, to have buckets of sea water in readiness. By this treatment he always procured relief; and his habits of living induced many severe accessions, almost every year, for which he would be carried into the sea and there remain until the accustomed alleviation of his symptoms took place; besides this, he had the inflamed parts wrapped in cloths immersed in the same fluid, which never failed of procuring the desired relief.

*St. George's Place, Blackfriar's Road,*
*April* 10, 1804. PERSCRUTATOR.

We request this Correspondent to send for his other paper, as some parts require elucidation. Ed.

